# A Coproantigen Diagnostic Test for *Strongyloides* Infection

**DOI:** 10.1371/journal.pntd.0000955

**Published:** 2011-02-08

**Authors:** Alex M. Sykes, James S. McCarthy

**Affiliations:** Clinical Tropical Medicine Laboratory, Queensland Institute of Medical Research, University of Queensland, Herston, Australia; Hospital Universitário, Brazil

## Abstract

Accurate diagnosis of infection with the parasite *Strongyloides stercoralis* is hampered by the low concentration of larvae in stool, rendering parasitological diagnosis insensitive. Even if the more sensitive agar plate culture method is used repeated stool sampling is necessary to achieve satisfactory sensitivity. In this manuscript we describe the development of a coproantigen ELISA for diagnosis of infection. Polyclonal rabbit antiserum was raised against *Strongyloides ratti* excretory/secretory (E/S) antigen and utilized to develop an antigen capture ELISA. The assay enabled detection of subpatent rodent *S. ratti* and human *S. stercoralis* infection. No cross-reactivity was observed with purified E/S from *Schistosoma japonicum*, the hookworms *Ancylostoma caninum*, *A. ceylanicum*, nor with fecal samples collected from rodents harboring *Trichuris muris* or *S. mansoni* infection. *Strongyloides* coproantigens that appear stable when frozen as formalin-extracted fecal supernatants stored at −20°C remained positive up to 270 days of storage, whereas supernatants stored at 4°C tested negative. These results indicate that diagnosis of human strongyloidiasis by detection of coproantigen is an approach worthy of further development.

## Author Summary

*Strongyloides stercoralis* is almost unique among human nematode infections in its ability to replicate within a patient's body, potentially leading to life-long infections if left untreated. Given the potential for severe life threatening *Strongyloides* infections and the unsatisfactory results of current parasitologic and antibody tests, there is a need for more efficient diagnostic tools. In this study we generated an assay to specifically detect proteins expelled by *Strongyloides*. Initially this assay for *Strongyloides* detection was not specific for the parasite; however, after developing a methodology using formaldehyde preservation of feces we specifically detected *Strongyloides* antigens in rodent and human stool. This methodology was then tested for cross-reactivity with purified proteins from closely related parasites and furthermore for cross-reactivity against faeces collected from animals harbouring single parasitic infections. Using this approach we found no non-specific reactivity with host or to various parasite antigens, suggesting that this assay is truly specific for *Strongyloides* detection.

## Introduction

The diagnosis of gastrointestinal infections typically relies on either empirical clinical diagnosis, or demonstration of the pathogen using standard microbiological techniques. However, a number of important gastrointestinal pathogens are difficult to detect using such techniques, and in some of these infections such as amoebiasis and strongyloidiasis, a potentially life-threatening pathogen may be present despite few or no clinical symptoms and negative diagnostic tests.

Serological diagnosis by ELISA [1], [2] and agar plate coproculture [1], [2] are currently the standard techniques used for parasitalogical diagnosis of infection with *Strongyloides stercoralis*. Agar plate coproculture has been reported to show high sensitivity and specificity. However, it requires a 24–48 hours incubation time, an experienced technician to correctly identify the larvae, and can represent a biohazard to the laboratory scientist [1], [2]. While serologic diagnosis, usually by ELISA is highly sensitive [3], specificity can be low due to the persistence of antibodies from previous infection or cross-reactive antibodies [4].

Coproantigen assays have been developed for the diagnosis of a range of human and animal intestinal infections. In general antibodies raised against whole parasite extracts are coated on microtiter plates, and subsequently fecal antigen is captured and detected with the same or second parasite-specific antibody in a capture assay [5], [6]. The use of monoclonal antibodies is generally believed to increase both the sensitivity and the specificity of such assays [7]–[10].

Examples of coproantigen ELISAs developed for the detection of a range of intestinal infections include amoebiasis [11]–[13], bacterial and viral gastroenteritis [14], trematodes [15] and cestodes [16], [17]. Of note, the more successful coproantigen assays have been developed against intestinal infections with either large parasites or pathogens with a likely high fecal antigen load. However, coproantigen detection is not limited to diagnosis of intestinal infections, as it has also been applied to the detection of gastric adenocarcinoma [18] and the detection of fecal occult blood [19]. Another advantage of such coproantigen assays is the ability to detect cryptic or pre-patent infections [6], [20].

In the case of nematode infections, a number of assays have been reported for the detection of animal host parasites, including *Necator americanus* [21], *Ancylostoma ceylanicum* [20], *Teladorsagia circumcincta* [22] and *Heligmosomoides polygyrus*, [6]. There have also been reports where assay sensitivity and/or specificity was unsatisfactory e.g. detection of *Strongyloies ratti* [23], *Haemonchus contortus* [24] and *Ostertagia ostertagi* [25]. With the exception of the *O. Ostertagia* assay, assays of satisfactory performance have employed specific antiserum raised against excretory/secretory (E/S) antigens of the respective parasites, rather than utilizing antiserum raised against total somatic antigen. However, the usefulness of these assays in the detection of heterologous antigen in human infections, or in the development of an assay to detect human nematode infection has not been reported.

In this study, polyclonal antiserum was raised against *S. Ratti* E/S antigens and used to develop an assay capable of detecting *Strongyloides* antigen present in the feces of rodents harbouring *S. Ratti*. This assay was then tested in a pilot proof of principle study in a human patient with patent *S. Stercoralis* infection. The sensitivity of the antibody for detection of *S. Ratti* antigen as well as heterologous *S. Stercoralis* coproantigen was investigated. Techniques to reduce cross-reactivity with fecal components were also explored, and the analytical specificity of the coproELISA was determined by testing E/S and fecal supernatants collected from other animals or humans with helminth infections. Finally, the effect of preservation and storage conditions of fecal samples and coproELISA was investigated.

## Materials and Methods

### *S. ratti* life cycle

The *S. ratti* life cycle was maintained in 4–8 week old male Wistar rats and infection was established as previously described [26]. Parasites to establish the life cycle were kindly provided by Prof. Mark Viney (University of Bristol, UK).

### E/S antigen preparation

E/S antigens for immunization was collected from parasitic adult worms harvested from infected rats 10–14 days post-infection and rinsed extensively in RPMI (Invitrogen, Carisbad, CA) containing 200 µg/ml ceftriaxone (Roche, Basel, Switzerland), 2.5 µg/ml Amphotericin B (Sigma-Aldrich, St. Louis, MO) and 400 µg/ml Gentamicin. Cleaned parasitic adult worms were incubated at 37°C in 5% CO_2_ for 24 hours in 3–4 ml of RPMI containing 20 µg/ml ceftriaxone, 0.25 µg/ml Amphotericin B and 40 µg/ml Gentamicin in 6 well tissue culture plates (Falcon). Immediately after incubation, a pre-mixed cocktail of protease inhibitors (complete, mini-protease inhibitor cocktail, Roche) was added to a 1X final concentration, and the E/S products stored at −20°C. Frozen E/S products were centrifuged at 3,000 g for 5 min and the supernatant collected. Concentration and dialysis of E/S was undertaken in Centricon YM-10 devices (Millipore, Billerica, MA) according to the manufacturer's instructions. Protein concentration was determined using a BCA kit (Thermo Scientific, Waltham, MA).

### E/S antigens collected from other helminths

*S. stercoralis* E/S products were kindly donated by Prof Gerhard Schad (School of Veterinary Medicine, University of Pennsylvania). Adult worms were collected from an experimentally infected hamster harboring ∼390 parasitic adult worms. E/S products were collected from these parasitic adult worms as previously described, and lyophilized. Lyophilized E/S antigens were reconstituted in distilled water, concentrated and quantitated as previously described for *S. ratti* E/S antigens. *Schistosoma japonicum* E/S products were collected from 100 pairs of *S. japonicum* adult worms that were cultured for 24 hours in RPMI. *A. caninum* E/S antigens were kindly provided by Tegan Don (Queensland Institute of Medical Research, Brisbane, Australia). Adult *A. caninum* worms were harvested from the small intestine of necropsied pound dogs. *A. ceylanicum* E/S products were donated by Prof Jerzy Benkhe (University of Nottingham, UK). Adult worms were harvested from experimentally infected hamsters. All protein preparations were concentrated, dialysed and quantitated as previously described for *S. ratti* E/S antigens.

Uninfected rat feces and fecal samples from *S. ratti*-infected rats were collected from laboratory rats housed at the Queensland Institute of Medical Research. Pooled feces collected from 6 mice without parasitic infection and 6 mice infected with *S. mansoni* was supplied by Mary Duke (Queensland Institute of Medical Research, Brisbane, Australia). Lyophilized *T. muris*-infected mouse fecal supernatant was supplied by Prof. Jerzy Benkhe (University of Nottingham, UK). Queensland Medical Laboratories (Brisbane, Australia) supplied anonymous uninfected human feces and three consecutive *S. stercoralis*-infected stool samples. These had been collected from a single patient with *S. stercoralis* infection proven by agar plate coproculture.

### Fecal supernatants

Fecal supernatants were prepared at a ratio of 1∶3 (v/v) in a solution of PBS-T (PBS containing 0.3% Tween-20), 4% formalin or 10% formalin, and vortexed to homogenise in a 50 ml conical tube. Samples were centrifuged at 3,200 g for 15 min at 4°C, after which supernatant was collected and centrifuged to remove fecal debris. The cleared supernatants were then aliquoted and stored at −20°C unless otherwise specified.

### Rabbit polyclonal anti-E/S antibody (α-E/S-Ab)

A New Zealand white rabbit was immunized with lyophilized *S. ratti* E/S products at the Institute for Medical and Veterinary Sciences (Adelaide, Australia). The rabbit was bled prior to immunization to provide a negative control. The primary immunization consisted of 400 µg E/S antigen emulsified in Freund's complete adjuvant, injected subcutaneously. The antibody response was boosted with 200 µg E/S antigen emulsified in Freund's incomplete adjuvant and injected sub-cutaneously. Terminal bleed serum was shipped overnight at 4°C to the laboratory prior to aliquotting and storage at −80°C.

### Purification of immunoglobulins

Protein A sepharose (GE Healthcare, Chalfont St. Giles, United Kingdom) was used to purify α-E/S immunoglobulin from serum according to the manufacturer's instructions. IgG was eluted from the Protein A column by the addition of 1 ml 0.1 M glycine (pH 3.0). The eluate was immediately neutralized in 60 µl 1 M Tris (pH 9.0) and separated into ten separate fractions. Optical density was monitored by spectrophotometry at a wavelength of 320 nm to identify fractions containing IgG. These were pooled and dialysed by four changes of PBS in Centriprep YM-50 concentrators (Millipore). Purified α-E/S Ig was quantitated by spectrophotometry where 1 mg/ml Ig  = A_320_ of 1.43 [27].

### Biotinylation of purified α-E/S immunoglobulins (α-E/S-B)

In a 1.5 ml tube, 1.63 mg of α-E/S Ig was diluted in 100 µl NHS-LC biotin working solution (0.1 mg NHS-LC biotin (Thermo Scientific) in 100 µl dimethylformamide). The volume brought up to 1.1 ml with PBS and biotinylated according to the manufacturer's instructions. Unbound biotin was removed, and the biotinylated anti-E/S antibody was dialysed by centrifugation in a 30 kDa nanosep spin column (Pall Life Sciences, USA) at 10,000 g at 4°C. Biotinylated α-E/S antibody was recovered from the spin column by washing the membrane with 500 µl PBS and collecting the retained immunoglobulins. Biotinylated α-E/S antibody was aliquotted and stored at −80°C. Biotinylation was confirmed by dot blot analysis of labelled immunogobulin.

### Coproantigen ELISA

The assay components were titrated by doubling dilution to determine optimal signal:noise ratios. 96 well flat bottom microtiter plates (Nunc, Thermo Scientific) were coated with 50 µl (5 µg/ml) α-E/S Ig diluted in coating buffer (15 mM Na_2_CO_3_, 35 mM NaHCO_3_, pH 9.6) and incubated overnight at 4°C or at room temperature for 90 min. After incubation, wells were washed 4–5 times in PBS-T (PBS containing 0.05% Tween20). Wells were then blocked with 150 µl of PBS containing 2% casein (Merck, San Diego, CA) for 60 minutes at room temperature. Uninfected rat fecal supernatant (nRFS) and infected fecal supernatants (iRFS) were diluted 1∶4 in PBS unless otherwise specified, and 50 µl added to each well and incubated for 90 min at room temperature. Known positive and negative formalin-fixed rat fecal supernatants, E/S products diluted in PBS and uninfected formalin-fixed fecal supernatant were assayed in duplicate. Wells containing only PBS or nRFS were included on each plate for standardization. Plates were then washed 4 times in PBS-T, after which 50 µl of 1∶500 α-E/S-B was added, and incubated at room temperature for 60 min. The plates were again washed 4 times in PBS-T and 50 µl of NeutrAvidin-HRP (Thermo Scientific) diluted 1∶10,000 was added, and the plates incubated for a further 60 min at room temperature. Plates were then washed 4 times in PBS-T prior to the addition of 100 µl per well of the developing substrate, ABTS (2,2′-azino-bis(3-ethylbenzthiazoline-6-sulphonic acid)). After incubation at room temperature for 20–30 min plates were read on a Versamax microplate reader (Molecular Devices) at 405 nm, and analysed with Prism 4 (Graphpad software, La Jolla, CA).

### Ethics

Approval for maintenance of the life cycle of *S. ratti* in laboratory rats was obtained from the Animal Ethics Committee of the Queensland Institute of Medical Research. Ethical approval to test anonymised, non-reidentifiable fecal samples from patients with known parasitologic status for strongyloides was granted by the Royal Brisbane and Womens Hospital Human Research Ethics Committee.

## Results

### Sensitivity of the coproantigen ELISA

**Figure 1:**
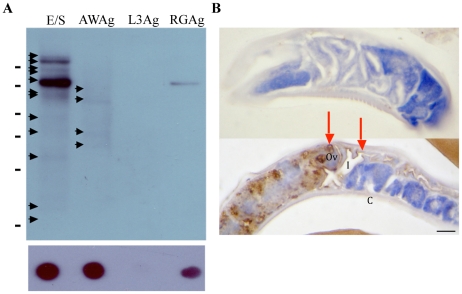
Immunoreactivity of the α-E/S antibody is primarily specific for adult *S. ratti* E/S. α-E/S IgG recognizes primarily *Strongyloides* E/S antigens (A): Western blot analysis of (E/S) adult worm E/S, (AWAg) adult worm antigen, (L3Ag) 3^rd^ stage larval antigen and (RGAg) soluble naïve rat gut antigen, using α-E/S IgG at a dilution of 5.5 µg/ml. A single immunodominant ∼75 kDa cross-reacting band was observed in the uninfected rat faecal supernatant immunoprecipitation experiment. Other immunoreactive protein bands are recognised by the α-E/S Ab and are not seen in uninfected rat gut extract. These *S. ratti*-specific proteins were detected at approximate molecular weights of 38 kDa, 40 kDa, 60 kDa and 70 kDa in AWAg, with additional 35 kDa, 50 kDa, 65 kDa, 100 kDa and >100 kDa bands being observed in E/S. (B) Immunoperoxidase-stained parasitic adult worm sections, probed with either (upper panel) 1∶500 preimmune rabbit serum, or (lower panel) α-ES IgG diluted 5.5 µg/ml in PBS. Scale bar  = 20 µm. Legend: I =  intestine; Ov  =  ovaries; C =  cuticle. Arrows highlight the specific ovarian and intestinal cells recognised by this antibody.

Polyclonal rabbit antiserum was initially raised against *S. ratti* E/S products (α-E/S Ab). This antiserum had an end titer of 1∶512,000 when measured by ELISA and was shown to recognise 14 protein bands on western blot (Figure 1A). Reactivity to adult worm antigen was lower, with only 4 protein bands recognised by western blot. Even less immunoreactivity was observed with infective 3^rd^ stage larvae where no reaction by immunoblot was observed (Figure 1A). We next characterised α-E/S Ab using immunohistochemistry to identify the target organs. This resulted in specific immuno-staining of the ovaries and intestine of the parasitic adult worm (Figure 1B). It was also apparent that during the production of E/S, that some contamination with rat host proteins had occurred, resulting in the generation of an antibody response to contaminating rodent host antigen (Figure 1A).

An antigen capture assay was developed using purified IgG of α-E/S Ab (α-E/S IgG) for the capture antibody; the same antibody now biotinylated was used for detection. To determine the lower limit of detection of this assay, *Strongyloides* E/S products were diluted in PBS from ≥2 µg/ml to ∼10 ng/ml by doubling dilution, and tested in the antigen-capture ELISA. The lower limit of E/S detection was determined to be 80 ng/ml for *S. ratt*i; the limit of detection for and heterologous *S. stercoralis* antigen was 500 ng/ml.

We next tested whether fecal components interfered with assay performance. PBS-T-extracted uninfected human fecal supernatant (nHFS), uninfected rat fecal supernatant (nRFS) and infected rat fecal supernatant (iRFS) were evaluated at concentrations ranging from undiluted to 1∶8 in PBS (data not shown). High background was observed in nRFS across all dilutions, making it impossible to discriminate between uninfected and infected samples. At the starting dilution, nHFS exhibited moderate assay background but became negative at a dilutions ≥1∶4.

Various techniques were evaluated in an attempt to reduce coproantigen ELISA cross-reactivity. These included a range of blocking agents, sample diluents, the addition of protease inhibitors, adsorption against host proteins and heat treatment. However, the majority of approaches had no effect on the background when tested with PBS-T-extracted feces. Likewise, changing the blocking agent from casein to bovine serum albumin, skim milk or fetal calf serum did not improve the assay performance. The addition of a commercial cocktail of protease inhibitors to fecal extracts did not decrease the background, suggesting proteases were not affecting the assay performance by desorbing protein coating the plate (data not shown). Pre-adsorption of cross-reactive antibodies using a cocktail of rat gut antigen and nRFS resulted in some reduction of cross-reactivity with host proteins but did not enable reliable distinction between infected and uininfected samples (data not shown).

**Figure 2:**
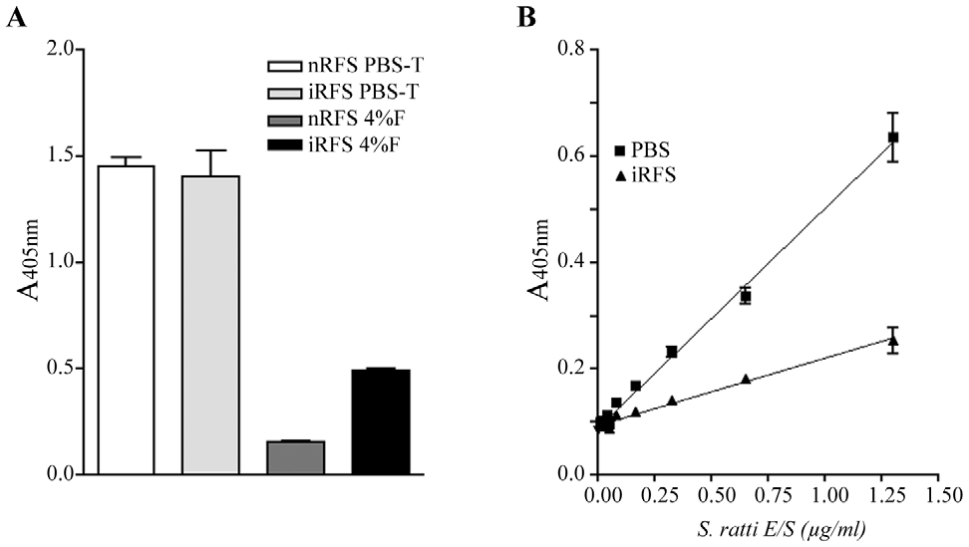
Formalin preservation allows specific discrimination of infected rat fecal supernatant from uninfected fecal supernatant. (A) Effect of assay specificity after various treatments. Pooled uninfected fecal supernatant collected from 6 rats (nRFS) and infected rat fecal supernatant collected from 2 rats infected with *S. ratti* (13 and 21 dpi) (iRFS) were extracted simultaneously in PBS-T and 4% formalin (4%F). Only extraction in formalin led to a positive discrimination between nRFS and iRFS. All points are mean ± SEM of OD values obtained from duplicate samples. (B) Detection of known amounts of E/S products diluted in PBS or uninfected rat fecal supernatant (nRFS) extracted in 4% formalin. The lowest concentration of E/S products detected was 80 ng/ml and 325 ng/ml when diluted in PBS and nRFS, respectively. All points are mean ± SEM of OD values obtained from 4 samples.

It was observed that changing the fecal extraction diluent from PBS to formalin resulted in a significant beneficial effect on assay background (Figure 2A), such that when fecal samples were extracted in formalin, a clear discrimination between uninfected and infected samples was apparent, with only infected rat feces testing positive. Again, the addition of FCS and heat treatment resulted in no significant improvement in assay performance. Using formalin extraction, the assay was sufficiently sensitive to detect infection in samples diluted at least eight-fold. However, assay sensitivity was reduced approximately 4 fold when E/S was diluted in formalin-extracted nRFS (Figure 2B).

### Specificity of coproantigen detection

**Figure 3:**
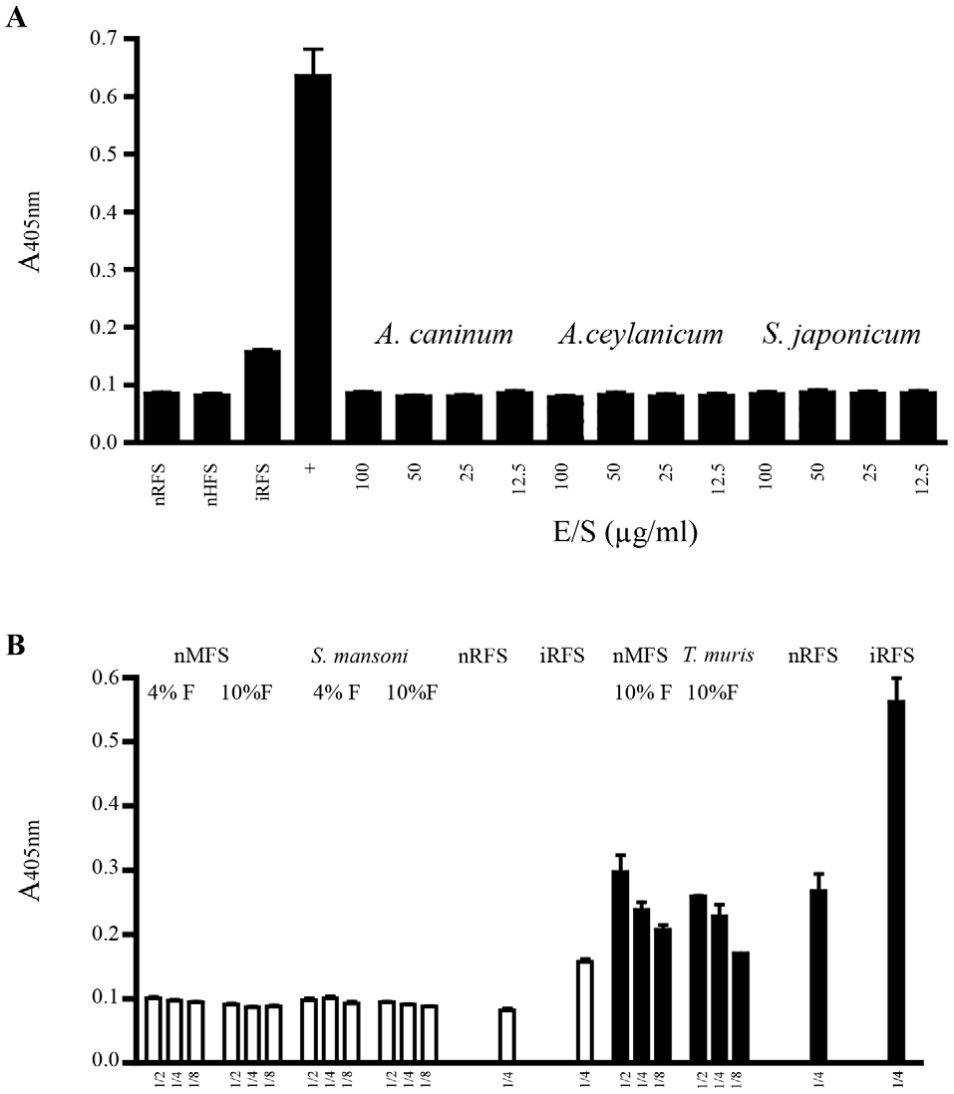
No cross reactivity is observed with purified E/S antigens or faecal supernatants collected from various helminths. (A) Testing of the *Strongyloides* coproELISA with E/S products collected from other helminths. All *Ancylostoma* and *Schistosoma* E/S products tested negative even at the high concentration of 100 µg/ml. Pooled uninfected rat fecal supernatant collected from 6 rats (nRFS) and infected rat fecal supernatant (iRFS) collected from two rats (13 and 21 days post infection). iRFS and 1.3 µg/ml *S.ratti* E/S (+) tested positive. All points are mean ± SEM of OD values obtained from 4 identical samples. (B) Specificity for *Strongyloides* coproantigen detection. White bars and black bars represent data from two different experiments. Diluted samples were prepared from undiluted fecal supernatant to 1∶4 by doubling dilution in PBS. Uninfected mouse fecal supernatant (nMFS), *Trichuris muris* and *Schistosoma mansoni* supernatants test negative even when applied undiluted. Uninfected rat fecal supernatant (nRFS) and infected rat fecal supernatant (iRFS) tests negative and positive, respectivly. All points are mean ± SEM of OD values obtained from duplicate samples. Assay cuttoff for negative reading is represented by the grey box.

We next assessed analytical specificity of the assay. Known amounts of E/S collected from *Ancylostoma caninum*, *A. ceylanicum* and *Schistosoma japonicum* adult worms were tested at concentrations ranging from 100 µg/ml to 12.5 µg/ml. No cross-reactivity was observed from the E/S of species of helminth tested, even at concentrations as high as 100 µg/ml (Figure 3A). Likewise, fecal supernatants from mice with monoparasitic infections *Trichuris muris* and *S. mansoni* which are more likely to co-infect humans and Formalin-fixed feces collected from uninfected human, tested negative (Figure 3B).

### Coproantigen present in fecal supernatants are not stable after storage at 4°C

**Figure 4:**
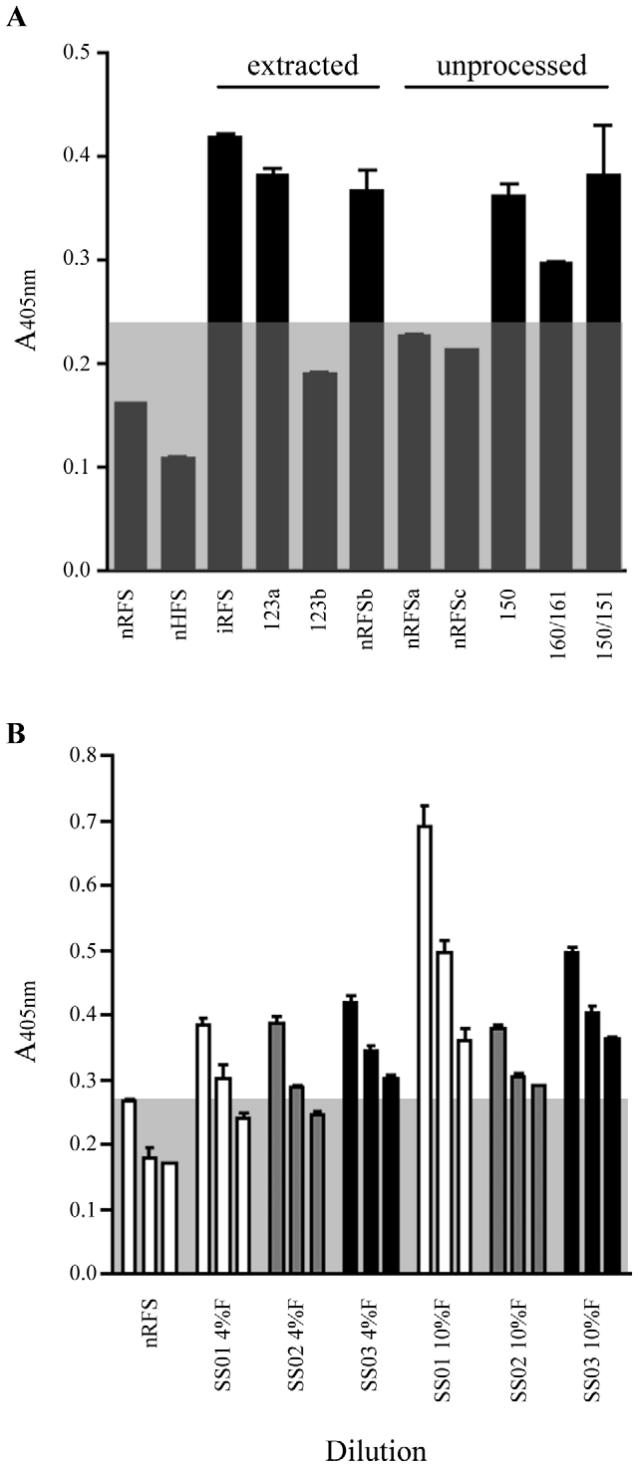
Effect of sample storage on rat fecal supernatant and detection of heterologous *S. stercoralis* coproantigen. (A) Pooled uninfected rat fecal supernatant collected from 6 rats (nRFS) and infected rat fecal supernatant (iRFS) collected from two rat (13 and 21 dpi) had previously been stored at −20°C and consistently tested negative and positive, respectively. Numbered samples 123, 150, 151, 160 and 161 represent samples collected from rats infected with known amounts of *S. ratti* infective larvae. Extracted fecal supernatant stored at 4°C displayed erratic absorbance values, as fecal supernatants from both uninfected and infected rats test positive. All unprocessed *S. ratti* infected feces stored in formalin (4% or 10%) tested positive for coproantigen when extracted after storage. Uninfected rat fecal feces tested negative when stored unextracted. All points are mean ± SEM of OD values obtained from duplicate samples. (B) Uninfected human fecal supernatant (nHFS) and *Strongyloides*-infected human fecal supernatants collected daily from a single patient with clinically diagnosed strongyloidiasis (SS01-SS03) were extracted simultaneously with 4% formalin and 10% formalin. All points are mean ± SEM. Assay cuttoff for negative reading is represented by the grey box.

To explore the stability of coproantigen for subsequent detection in *S. ratti-*infected rat feces, the effect of storage on fecal supernatants was investigated. Stability was investigated by incubating unprocessed feces stored in formalin and processed feces as formalin-extracted fecal supernatant at 4°C for various lengths of time. Other formalin-extracted fecal supernatants were stored in aliquots at −20°C. Frozen formalin-extracted nRFS pooled from 6 rats consistently tested negative. In contrast, frozen formalin-extracted iRFS consistently tested positive by coproELISA (Figure 4A).

The effect of prolonged storage at 4°C of formalin-extracted fecal supernatant, and unprocessed formalin-preserved *S. ratti* rat feces for testing by coproantigen ELISA is shown in Figure 5. When formalin-extracted fecal supernatants were tested, both uninfected and infected rat feces extracted in 10% formalin tested negative. Feces collected simultaneously from both uninfected rats and *S. ratti*-infected rats, tested positive when extracted in 4% formalin, indicating that some assay cross-reactivity occurred at sub-optimal concentration of formalin. In contrast, *S. ratti*-infected rat feces stored unprocessed in formalin tested positive when extracted as fecal supernatant, whereas uninfected rat feces stored unprocessed in formalin consistently tested negative even after 270 days at 4°C.

### *S. stercoralis* coproantigen can be detected human infection

Fecal samples collected from a human subject with proven *S. stercoralis* infection diagnosed by the agar plate method were tested next. Three separate samples were processed within 24 hours of collection. One gram of each stool was subject to concentration by sodium nitrate flotation for parasitological diagnosis of infection [28]. In addition, approximately 20% of the first and second stool samples were tested by Baermann coproculture. All 3 samples tested negative in 8 coverslips of sodium nitrate-floated feces for the presence of *Strongyloides* and all samples tested negative using the Baermann migration test.

Figure 4B shows the results of the assay in which the three formalin-extracted human samples were tested with coproantigen ELISA from dilutions of 1∶2 to 1∶8. In this experiment, a 1∶4 dilution of formalin-fixed nHFS was used as the negative control and the cut-off threshold for the assay determined. Uninfected human feces extracted in 10% formalin yielded a positive result at the assay cut-off when assayed at a 1∶2 dilution, but remained negative at dilutions of 1∶4 and below. The first and second samples tested positive at dilutions of 1∶2 and 1∶4, while sample the third tested positive at all dilutions. When extracted in 10% formalin, all test samples tested positive for coproantigen, even when diluted 1∶8.

## Discussion

Prior to this study, there had been one report describing coproantigen detection for *S. ratti*. In this work, antiserum was raised against whole worm antigen of *S. ratti* larvae and parasitic adult worms [23]. However, the authors reported low signal:noise ratios for positive fecal samples and minor cross-reactivity with the nematodes *Syphacia muris* and *Necator americanus*. The observation of high assay background is likely due to contamination of the antigen preparation with host gut and fecal material. Thus, the assay was not considered sufficiently sensitive for use as a diagnostic test [23]. More recently coproantigen tests have used antiserum raised to E/S antigens for the diagnosis of animal nematode infections. In general this approach has proved to be more useful [6], [20], [22].

An objective of this study was to determine whether heterologous *S. stercoralis* coproantigen could be detected in an infected human with antiserum raised to rodent *Strongyloides* E/S antigens. Data supporting this approach included studies documenting cross-reactivity between species with various published serodiagnostic and coproantigen assays [4], [21], [29], [30]. Although, there have been numerous reports of the detection of coproantigen in animal nematode infections [6], [20], [22], to our knowledge this is the first report that heterologous human infection has been detected by coproELISA with antiserum raised to antigens from an animal nematode.

The anti-*Strongyloides* E/S polyclonal antibody was observed to cross-react with contaminating rat gut proteins present in rat feces. This cross-reactivity resulted in false positive tests when fecal supernatants collected from control rats were tested by coproELISA. Cross-reactivity with fecal components is a common occurrence in coproantigen assays and many techniques have been published describing how to reduce cross-reactivity [6], [22], [31], [32]. Published methods for reducing background in coproELISAs are aimed at improving specificity by adsorbing cross-reactive antibodies, optimising method for extraction of the target antigen including the diluent used for fecal supernatant, or the choice of assay blocking agent. A range of these methods was tested with the *Strongyloides* α-E/S indirect coproELISA. However, the only method that reliably reduced assay background while preserving a positive signal in infected feces was formalin extraction of fecal supernatants. A possible explanation for this is that formalin treatment resulted in cross-linking the epitopes of the cross-reactive antigens in such a way that the non-specific antibodies in α-E/S Ab no longer bound. An alternative hypothesis is that the host antigens are fixed to host debris that are removed by centrifugation. Coproantigen detection in formalin-extracted fecal supernatant has also been successfully applied for diagnosis of *Giardia* [33], *Echinococcus* [8], [29], *Taenia* [34] and *Cryptosporidium* [35].

An important consideration in the *Strongyloides* coproantigen ELISA is the use of formalin-extracted fecal supernatant for the discrimination between uninfected and infected samples as formalin exerts its effect by protein cross-linking. Thus, the sensitivity of E/S product detection in formalin-extracted feces could be impaired. This hypothesis was confirmed in an experiment where purified *S. ratti* E/S was diluted in PBS and formalin fixed uninfected faecal supernatant. An additional advantage of this preservation method is that it removes potential biohazards. Dilution of E/S in unfixed faecal supernatant has also been demonstrated to decrease assay signal:noise ratio and may be a contributing factor in the decreased sensitivity [6], [20], [33].

Even though the sensitivity for detection of *S. stercoralis* E/S (0.5 µg/ml) was approximately 6 fold less than the detection limit for *S. ratti* E/S (0.08 µg/ml). The sensitivity of nematode coproELISAs currently range from 0.01 µg/ml to 0.5 µg/ml E/S [6], [20], [22], which is in agreement with the observed sensitivity of the *Strongyloides* coproELISA.

An important hurdle in diagnostic assays for parasitic infections is cross-reactivity with antibodies or antigens from heterologous parasites, a major issue described in many nematode coproantigen and serodiagnostic assays including current *Strongyloides* antibody tests [22], [36]. No cross reactivity was observed in experiments with E/S antigens from *S. japonicum*, murine *S. mansoni* fecal supernatants or samples from the more closely related nematodes *A. caninum* and *A. ceylanicum*, or fecal supernatants from mice harboring *T. muris* infection. Together, these experiments suggest that the assay is specifically detecting *Strongyloides* antigens, as no cross-reactivity was noted with the other helminths tested.

The last and most important investigation of the *Strongyloides* coproantigen ELISA involved testing *S. stercoralis-*infected and -uninfected human fecal samples. Importantly all *S. stercoralis-*infected samples extracted in 4% or 10% formalin tested positive when assayed at the standard sample dilution of 1∶4. Two fecal supernatants extracted in 4% formalin tested negative when diluted further to 1∶8. However, when simultaneously extracted in 10% formalin the samples tested positive. This suggests that this assay is more robust when fecal supernatant is extracted at the common fixative concentration, 10% formalin. This finding is similar to that reported by [33]where fixation in 10% formalin increased ELISA values in comparison with ELISA values obtained with distilled water extracted supernatants.

Finally, the stability of coproantigen after prolonged storage was addressed. A few reports have examined the apparent stability of coproantigen after prolonged storage at 4°C and −80°C, as well as following environmental desiccation of fecal samples [6], [20], [37]. The results of the data from this study suggest that antigens present in formalin-extracted fecal supernatants are not stable when left at 4°C, but were so when stored at −20°C. Interestingly, when infected feces were stored in formalin at 4°C for at least 6 months prior to fecal supernatant extraction they tested positive. This suggests that the coproantigens are stable when feces are preserved in formalin and stored at 4°C, but are not stable when fecal supernatant was extracted prior to refrigerated storage. These results contrast to a similar study where formalin extracted and water extracted faecal supernatants were both stable at 4°C for the detection of *Giardia* cyst antigens [33].

The *Strongyloides* antigens detected by the coproELISA are currently undefined and future work in identifying the actual antigen(s) and raising monoclonal antibodies should enhance assay sensitivity and eliminate host cross-reactivity. The efficacy of the coproELISA is likely to be greatly enhanced if host cross-reactivity is eliminated and unfixed samples could also be assessed, broadening the utility. Another advantage of developing the *Strongyloides* coproantigen monoclonal antibodies would be in the conversion of the coproELISA into a rapid immunochromatographic dipstick test, a number of which are commercially available [38]. Further studies with a wider population of patients harbouring a variety of monoparasitic infections will also be required to accurately determine the specificity and sensitivity of the coproELISA.

In conclusion, we have developed a coproELISA for the detection of strongyloidiasis, and have demonstrated that coproELISA using antibodies raised against antigens of a related animal parasite can be used to diagnose human infection in an antigen capture ELISA. Furthermore, this is the first report of an antigen capture assay using antibodies raised against native *Strongyloides* E/S antigens. Unlike many published assays where fecal supernatant is extracted in PBS containing a non-ionic detergent [6], [16], [20], [22], [29], the current assay was only effective in discriminating uninfected from *Strongyloides*-infected fecal supernatant when extracted from formalin-treated feces. Future work with this assay will lead to a better diagnosis of human infection and enhanced understanding of the biochemistry of *Strongyloides* infections.
